# Case Report: A case analysis of antitumor therapy and cardiovascular safety management in a breast cancer patient with multiple cardiovascular diseases

**DOI:** 10.3389/fcvm.2026.1690244

**Published:** 2026-04-28

**Authors:** Minghui Long, Boni Ding

**Affiliations:** Department of Breast and Thyroid Surgery, Third Xiangya Hospital, Central South University, Changsha, China

**Keywords:** antithrombotic bridging, breast cancer, cancer therapy-related cardiovascular toxicity (CTR-CVT), cardiovascular toxicity, multidisciplinary management

## Abstract

**Background:**

As breast cancer treatment advances, therapeutic efficacy has improved significantly, but cardiovascular toxicity from anticancer agents has become a critical concern. Elderly patients often have pre-existing cardiovascular comorbidities like coronary heart disease (CHD) and high venous thromboembolism (VTE) risk, requiring long-term antithrombotic therapy. Perioperative management demands individualized assessment of thrombotic and bleeding risks, with bridging anticoagulation when necessary. Close surveillance for cardiovascular complications (myocardial injury, heart failure, hypertension [HTN]) and control of modifiable risk factors (HTN, diabetes mellitus [DM]) are essential during treatment.

**Case report:**

This study presents a breast cancer patient with multiple cardiovascular comorbidities (HTN, DM, CHD). Following multidisciplinary team (MDT) consultation, the low-cardiotoxicity PTD regimen (pertuzumab, trastuzumab, docetaxel) was selected. Perioperatively, aspirin was withheld, and low-molecular-weight heparin (LMWH) bridging was used 24 h before and after radical mastectomy. Postoperative radiotherapy was administered, with regular cardiac function monitoring throughout treatment. No cardiovascular complications occurred.

**Conclusion:**

For cancer patients with cardiovascular comorbidities, balancing tumor treatment and cardiovascular safety is achievable through comprehensive baseline risk assessment, the selection of low-cardiotoxicity regimens, rational perioperative antithrombotic adjustments, and continuous cardiac monitoring, thereby ensuring optimal oncological outcomes with minimized cardiovascular risks.

## Introduction

Cancer Therapy-Related Cardiovascular Toxicity (CTR-CVT) encompasses a broad spectrum of cardiac functional alterations induced by various anticancer treatments, including chemotherapy, targeted therapy, immunotherapy, and radiotherapy. These alterations include cancer therapy-related cardiac dysfunction (CTRCD), coronary artery disease (CAD), valvular heart disease, arrhythmias, hypertension, thrombosis and thromboembolic events, peripheral artery disease, bleeding complications, pulmonary hypertension, and pericardial diseases. The 2022 ESC Guidelines on Cardio-Oncology systematically proposed risk stratification, baseline assessment, monitoring pathways, and long-term follow-up strategies for CTR-CVT (1), emphasizing that high-risk patients should undergo comprehensive cardiovascular evaluation and dynamic monitoring to balance therapeutic efficacy and safety. A multidisciplinary team (MDT), integrating expertise from oncology, cardiology, imaging, and radiation oncology, plays a pivotal role in treatment selection (e.g., avoiding anthracyclines, optimizing targeted therapy combinations), perioperative antithrombotic management, and the monitoring and intervention of cardiac toxicity. For patients with HER2-positive breast cancer and concomitant multiple cardiovascular diseases, ensuring treatment efficacy while reducing cardiovascular complications has become a critical clinical challenge.

This article reports a case of a patient with HER2-positive breast cancer and multiple cardiovascular risk factors. Through individualized treatment and rigorous monitoring guided by an MDT, a successful balance between cancer therapy and cardiovascular safety was achieved, aiming to provide a reference for clinical practice.

## Case report

A 62-year-old female with a history of hypertension (HTN), diabetes mellitus (DM), and coronary heart disease (CHD) was admitted in November 2023 due to multiple left breast masses. She had undergone percutaneous coronary intervention (PCI) to the left anterior descending artery(LAD) in March 2019 and was on long-term medications including valsartan, metformin, clopidogrel, and rosuvastatin. Physical examination revealed hard, grape-sized masses in the left breast (≈2 cm) without skin changes or nipple discharge. Axillary lymph nodes were non-enlarged.

Laboratory tests revealed elevated triglycerides (3.27 mmol/L) and blood glucose (13.32 mmol/L), but normal liver/kidney function, as well as normal levels of BNP and cardiac troponin. Electrocardiogram indicated sinus rhythm with QS waves in V2-V3 and flattened T-wave in lead I. Echocardiography revealed a left ventricular ejection fraction (LVEF) of 69%, mild septal thickening, reduced left ventricular compliance, and mild valvular regurgitation. Mammography, ultrasound, and MRI classified the left breast lesions as BI-RADS 5 with axillary lymph node metastasis. Core biopsy confirmed grade II invasive ductal carcinoma with ER/PR negativity, HER2 overexpression, and Ki-67 of ≈20%.

Given her high CTR-CVT risk (age, HTN, DM, CHD post-PCI), MDT recommended neoadjuvant therapy with the PTD regimen (docetaxel, trastuzumab, pertuzumab) to avoid anthracycline-trastuzumab combination, which increases heart failure risk (16% vs. 1%–3%). Perioperative antithrombotic management: clopidogrel was discontinued 5 days before surgery. We used LMWH bridging therapy based on PRECISE-DAPT and HAS-BLED scores (low bleeding risk). After 6 cycles, MRI showed reduced tumor and lymph node size. She underwent modified radical mastectomy on March 11, 2024, with postoperative pathology showing no residual invasive cancer and 1/16 positive axillary nodes. Postoperative radiotherapy (50Gy/25F) was administered with strict heart dose constraints (mean heart dose <8 Gy). Throughout treatment, regular monitoring included blood tests, cardiac biomarkers, ECG, and echocardiography. Post-treatment LVEF was 59%, with no cardiovascular complications. Key management principles included baseline cardiovascular risk stratification using biomarkers, ECG, and echocardiography (LVEF and global longitudinal strain). Dynamic LVEF monitoring every 3 months identified subclinical injury early. For perioperative care, balancing thrombosis and bleeding risks guided LMWH bridging, with early resumption of antiplatelets (clopidogrel 600 mg loading dose) post-surgery.

## Discussion

### Perioperative antithrombotic management

For patients with post-PCI coronary artery disease (CAD) undergoing noncardiac surgery, balancing thrombotic and bleeding risks is critical. This patient, 4 years post-PCI (>12 months), had low thrombosis risk, meeting elective surgery criteria (1). Stent thrombosis risk peaks 4–6 weeks post-PCI and remains elevated for up to 6 months. The recommended surgical time varies depending on the type of stent: ≥2 weeks for balloon dilation, ≥1 month for bare-metal stents/drug-coated balloons, ≥3 months for next-generation drug-eluting stents, and ≥12 months for biodegradable stents (2).

Using PRECISE-DAPT and HAS-BLED scores (3, 4), the patient's low bleeding risk (no bleeding history, normal liver/kidney function) supported safe LMWH bridging. Antiplatelet drugs act within 1 h, so oral therapy is resumed postoperatively. For post-PCI patients, P2Y12 inhibitors (e.g., clopidogrel 600 mg loading dose) should be restarted 24–48 h after surgery, with multidisciplinary assessment guiding interruption timing (2).

### Cardiovascular toxicity of antitumor therapy

The patient had HER-2-positive breast cancer with comorbidities including hypertension, type 2 diabetes mellitus, and coronary heart disease (post-PCI status), and was scheduled to receive anti-HER-2 targeted therapy. According to the 2022 ESC Guidelines on Cardio-Oncology, these comorbidities are all classified as risk factors for cardiovascular toxicity. Based on a comprehensive assessment using the Heart Failure Association/International Cardio-Oncology Society (HFA/ICOS) risk stratification tool (5), which evaluates patient-related factors (age, cardiovascular history, risk factors), cancer therapy-related factors (regimen and cumulative dose), and baseline cardiac assessments (electrocardiogram, echocardiography, cardiac biomarkers such as troponin and natriuretic peptides), this patient was classified as high-risk for cancer therapy-related cardiovascular toxicity (CTRCD).

The risk of cardiovascular toxicity is dynamic and requires sequential assessment before, during, and after cancer therapy. The primary goal is early identification of subclinical cardiovascular injury, enabling intervention before irreversible cardiac dysfunction develops, while avoiding unnecessary interruptions or discontinuations of anticancer therapy due to cardiovascular toxicity. In accordance with the consensus on managing high-risk patients, comprehensive baseline cardiovascular evaluations were completed prior to treatment initiation. These included obtaining a detailed cardiovascular history (symptoms, medications, family history), documenting treatment-related information (drug type, dosage, radiotherapy plan), performing a physical examination (blood pressure, heart rate, jugular venous distension, lower extremity edema), conducting laboratory tests (cardiac troponin cTnI/cTnT, NT-proBNP, complete blood count, liver and kidney function, electrolytes), and acquiring an electrocardiogram and echocardiogram. During treatment, echocardiography, cardiac biomarkers (cTn, NT-proBNP), electrocardiogram, and clinical symptoms were assessed regularly every 2–3 cycles. Early after treatment completion (at 3, 6, and 12 months), comprehensive cardiac function assessments, biomarker reassessments, and evaluations of cardiovascular risk factor control were performed. For such high-risk patients, lifelong follow-up is warranted, including annual echocardiography, biomarker monitoring every 6–12 months, and strict control of risk factors (blood pressure <130/80 mmHg, HbA1c < 7%).

Although the patient's LVEF decreased from 69% to 59%, a reduction of approximately 10%, she remained asymptomatic. This scenario introduces the concept of “permissible cardiotoxicity” (6). For patients at high or very high risk of CTRCD, continuing anticancer therapy may be acceptable under conditions of enhanced monitoring and cardioprotective support, provided the anticipated oncological benefit outweighs the cardiovascular risk. This approach requires three conditions: 1) a high expected benefit from cancer therapy (the patient achieved a pathological complete response); 2) absence of clinical heart failure symptoms; and 3) implementation of rigorous monitoring and a cardioprotective strategy (as demonstrated in this case through baseline assessment before treatment, dynamic monitoring every 3 months during treatment, and long-term follow-up after treatment).

Guidelines recommend the sequential regimen of EC followed by taxane plus targeted therapy as a first-line option for neoadjuvant treatment of HER-2-positive breast cancer. However, anthracyclines (e.g., doxorubicin, epirubicin) can cause dose-dependent, often irreversible, cardiotoxicity through mechanisms including topoisomerase II*β* inhibition, induction of oxidative stress, and mitochondrial dysfunction. The risk of heart failure increases with cumulative anthracycline dose, reaching 5%–26% at doses exceeding 550 mg/m^2^ (7–9). Trastuzumab, a monoclonal antibody targeting HER2, is associated with cardiac dysfunction that is typically reversible (Type II), characterized by myocardial stunning and contractile dysfunction without overt myocyte loss. The mechanism involves blocking the HER2 signaling pathway, which is crucial for cardiomyocyte survival and stress response. Notably, the sequential use of anthracyclines followed by trastuzumab synergistically increases the risk of heart failure. A study involving 286 patients with HER-2 positive breast cancer reported a CTRCD incidence of 4.5%, with all CTRCD cases having prior epirubicin exposure; the incidence of CTRCD was significantly higher in patients receiving both trastuzumab and pertuzumab (*P* = 0.003), identifying pertuzumab as an independent predictor (10). Importantly, systolic dysfunction was reversible in all patients, with a mean recovery time of approximately 4 months. This further underscores the importance of avoiding sequential anthracycline-trastuzumab regimens in high-risk patients. The choice of the PTD regimen (docetaxel, trastuzumab, pertuzumab) by the multidisciplinary team (MDT) in this case was based on this evidence, aiming to maximize the efficacy of dual HER2 blockade while minimizing synergistic toxicity. The patient achieving a pathological complete response confirms that this cardioprotective strategy did not compromise anticancer efficacy.

Radiotherapy-induced cardiovascular toxicity is a growing concern, particularly for patients with left-sided breast cancer. The landmark study by Darby et al. demonstrated that the risk of major coronary events increases linearly by 7.4% per 1 Gy increase in mean heart dose, with no apparent threshold (11). In this case, adjuvant radiotherapy (50 Gy in 25 fractions) was carefully planned, strictly adhering to dose constraints to keep the mean heart dose below 8 Gy, a threshold shown to significantly reduce the risk of LVEF dysfunction following radiotherapy in left-sided breast cancer patients.

Statins have emerged as a promising pharmacological intervention for preventing trastuzumab-related cardiotoxicity. Statins are commonly used cholesterol-lowering medications, and studies have indicated that their use may reduce the risk of heart failure in breast cancer patients treated with anthracyclines or trastuzumab (12). A 2024 umbrella review, which comprehensively analyzed 10 systematic reviews evaluating cardioprotective measures during trastuzumab therapy, found that among all interventions assessed, statins demonstrated the strongest cardioprotective effect, potentially preventing more than half of cardiac events during trastuzumab treatment, an effect superior to that of beta-blockers (13). This patient was already on rosuvastatin for secondary prevention of coronary heart disease. Continuing statin therapy during cancer treatment likely contributed to mitigating the incidence of cardiovascular toxicity associated with anti-HER-2 targeted therapy.

This case strongly demonstrates that for high-risk patients, a successful balance between optimal cancer therapy and cardiovascular safety can be achieved through multidisciplinary collaboration, individualized regimen selection, appropriate antithrombotic management, stringent radiotherapy dose control, vigilant cardiac function monitoring, and appropriate cardioprotective interventions.

## Data Availability

The original contributions presented in the study are included in the article/Supplementary Material, further inquiries can be directed to the corresponding author.
